# Mendelian randomization suggests that head circumference, but not birth weight and length, associates with intelligence

**DOI:** 10.1002/brb3.2183

**Published:** 2021-05-10

**Authors:** Li Qian, Fengjie Gao, Bin Yan, Lihong Yang, Wei Wang, Ling Bai, Xiancang Ma, Jian Yang

**Affiliations:** ^1^ Department of Psychological Medicine The First Affiliated Hospital of Xi’an Jiaotong University Xi'an China; ^2^ Clinical Research Center The First Affiliated Hospital of Xi’an Jiaotong University Xi'an China

**Keywords:** birth length, birth weight, infant head circumference, intelligence, Mendelian randomization

## Abstract

**Introduction:**

Birth parameters have long been reported to have a role in human intelligence. However, the causalities reported in previous observational studies were controversial. Our study aims to provide an unbiased investigation of the causal associations between birth parameters and human intelligence using the Mendelian randomization (MR) approach.

**Methods:**

Genetic instrumental variables for MR analyses were extracted from large genome‐wide association studies of infant head circumference (*N* = 10,768), birth length (*N* = 28,489), and birth weight (*N* = 321,223). Data for intelligence were obtained from a meta‐analysis of genome‐wide association studies of 269,867 individuals of the European ancestry. Primary MR analysis was performed using the standard inverse‐variance weighted method, and sensitivity analyses were performed using the weighted median, MR‐Egger, and MR‐PRESSO methods.

**Results:**

Using 10 single nucleotide polymorphisms as instrumental variables, we found that 1 standard deviation increase in infant head circumference was associated with 0.14‐fold higher scores in intelligence tests (β = 0.14, 95% confidence interval: 0.09 to 0.18, P_IVW_=2.05 × 10^–9^). The causal relationship was robust when sensitivity analyses were performed. However, birth length and birth weight had no significant associations with intelligence.

**Conclusion:**

Our findings suggested infant head circumference, but not birth weight and length were associated with intelligence, which might indicate that brain development rather than general fetal growth was responsible for the development of intelligence.

## Graphical abstract

We performed a Mendelian randomization study to investigate the causal effects of infant head circumference, birth length, and birth weight on human intelligence by extracting genetic association datasets from large‐scale genome‐wide association studies of birth parameters and human intelligence. Our findings suggest that that head circumference, but not birth weight and length, is associated with intelligence, which might indicate that that brain development rather than general fetal growth was responsible for the development of intelligence.

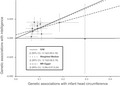

## 1 INTRODUCTION

Intelligence can be defined as one's ability to learn, imagine, understand complex ideas, engage in various forms of reasoning, and overcome obstacles by employing thought processes (Deary et al., 2010). Intelligence predicts important life and health outcomes, including school and career achievements (Deary et al., 2007), job performance (Burks et al., 2009), economic preferences (Strenze, 2007), health status (Gottfredson, 1997), and even expectation of death (Batty et al., 2008; Deary, 2008). Understanding the nature of individual differences in human intelligence has been an enduring goal for a wide range of psychologists.

The influence of birth parameters on intelligence is a contentious topic that has been widely discussed by physiologists and psychologists (Eide et al., 2007; Flensborg‐Madsen & Mortensen, 2017; Kormos et al., 2014). Broekman et al. declared that even small differences in birth parameters could be a sign of alterations in fetal development (Broekman et al., 2009). Over the past decades, numerous epidemiologic studies have reported significant relationships between intelligence and birth length (Eide et al., 2007), birth weight (Kristensen et al., 2014; Tong et al., 2006), and infant head circumference (Bach et al., 2020; Jaekel et al., 2019; Sammallahti et al., 2014). However, the results of these studies were inconsistent. For example, Galt et al. found no associations between head circumference and intelligence tests in a long‐term cohort study of 211 participants (Gale et al., 2004). Pearce et al. reported non‐significant results after a study of the effects of birth weight and birth length on some dimensions of cognitive function measurement (Pearce et al., 2014). So far, no consensus has been reached on the relationships between birth parameters and intelligence. A major reason for this may be that previous observational cohort studies were easily limited by multifarious confounders, such as pattern of infant feeding, environment, family education, and social experience (Boyko, 2013). Therefore, novel study designs that provide unbiased estimates of the causal associations between birth parameters and human intelligence were urgently needed.

Mendelian randomization (MR) is a newly developed genetic epidemiology approach that utilizes genetic variants extracted from genome‐wide association studies (GWASs) as instrumental variables to investigate the causal relationship between exposure and outcome of interest (Smith & Hemani, 2014). The fundamental assumption of MR study design is that if genetic architectures could predict the biological effects or level of an exposure, it should be also associated with the exposure‐related disease risk. Exploiting the fact that genotypes are not generally susceptible to reverse causation and confounding, the MR has the ability to provide unbiased estimate of causal relationship between the exposure and outcome (Lawlor, 2016). In recent years, MR has been widely applied to infer causal associations between various factors due to explosion in the availability of GWAS summary data (Haycock et al., 2017; Sanna et al., 2019; White et al., 2016; Yang et al., 2020). The aim of our study was to provide an unbiased investigation of the causal effects of infant head circumference, birth length, and birth weight on human intelligence by extracting data from GWASs of birth parameters and intelligence.

## 2 MATERIALS AND METHODS

### 2.1 Genetic instruments

Summary data of GWASs of infant head circumference (Taal et al., 2012), birth length (Valk et al., 2015), and birth weight (Warrington et al., 2019) were downloaded from the Early Growth Genetics Consortium (http://eggconsortium.org). We extracted 10 independent single nucleotide polymorphisms (SNPs) that were strongly (*p* < 1 × 10^−5^) associated with infant head circumference from the GWAS (*N* = 10,768) by Taal et al. (Table 1) (Taal et al., 2012). The 10 SNPs could explain approximately 2.6% of the variance in infant head circumference; the *F* statistic, another parameter used to evaluate the strength of the generated instrumental variable, was 28.6. For birth length, 21 independent SNPs (Table S1) at a significant threshold of *p* < 1 × 10^−5^ were extracted from the GWAS (*N* = 28,489) by Valk et al. (Valk et al., 2015). The 21 SNPs explained 2.1% of the variance in birth length, and the *F* statistic was 28.7. For birth weight, the GWAS (*N* = 321,223) by Warrington et al. reported 146 (of which 8 were not available in the GWAS summary dataset of intelligence) independent SNPs (Table S1) at a significance threshold of *p* < 6.6 × 10^−9^ (Warrington et al., 2019). The remaining 138 SNPs explained 2.8% of the variance in birth weight, and the *F* statistic was 67.0. All measurements, including infant head circumference, birth length, and birth weight, were carried out at birth or in infancy (6–30 months) according to standardized procedures and data were standardized (data were transformed into sex‐ and age‐adjusted standard deviation scores) using Growth Analyser (http://www.growthanalyser.org). The percentage of variance explained (R^2^) was calculated using the formula 2×minor allele frequency × (1‐minor allele frequency) × (β estimate)^2^, whereas the *F* statistic could be calculated from the R^2^ statistic as *F* = (N‐K‐1)/K × R^2^/(1‐R^2^), where N is the sample size and K is the number of SNPs (Burgess et al., 2016). Typically, an *F* statistic >10 is recommended for MR analyses (Burgess et al., 2013).

**TABLE 1:** Genetic determinants of infant head circumference and their associations with intelligence

SNPs	Gene	CHR	EA	EAF	Association with infant head circumference		Association with intelligence	
					β	*p* value	β	*p* value
rs11683142	LOC107985825	2	A	0.017	0.293	6.08e−06	−0.001	0.977
rs3094072	HLA‐L	6	C	0.160	0.104	1.52e−06	0.019	5.31e−06
rs1385504	COLEC10	8	A	0.141	0.094	7.08e−06	0.013	1.34e−03
rs1042725	HMGA2	12	T	0.490	−0.071	6.58e−10	−0.009	1.14e−03
rs7980687	SBNO1	12	A	0.200	0.091	3.35e−09	0.016	1.44e−06
rs12438760	‐	15	T	0.881	0.109	2.06e−06	0.006	0.194
rs9940645	ZNF423	16	A	0.442	0.066	5.88e−06	0.002	0.439
rs11655470	CRHR1	17	T	0.427	0.070	1.43e−06	0.013	1.32e−05
rs9675157	‐	17	C	0.057	0.185	9.61e−06	0.021	4.89e−04
rs238150	DDX27	20	T	0.780	−0.078	4.53e−06	−0.021	4.42e−10

### 2.2 GWAS of intelligence

We obtained data on genetic associations with human intelligence from a large‐scale meta‐analysis of GWASs of 14 cohorts, which were comprised of 269,867 individuals of European ancestry (Savage et al., 2018). Intelligence was assessed using various neurocognitive tests for different cohorts, but they were all operationalized to index a common Spearman's g factor of intelligence. Genotyping was performed separately in the 14 cohorts using either Affymetrix or Illumina SNP arrays. Stringent quality control procedures were applied to the summary statistics for each cohort before combining them for meta‐analysis. Finally, a total of 9,295,118 SNPs were included in the meta‐analysis. Further details on imputation, quality control, and association analysis can be found in the previously published study (Savage et al., 2018). Full GWAS summary statistics are publicly available at the Complex Trait Genetics lab website (https://ctg.cncr.nl).

### 2.3 Statistical analysis

Firstly, we extracted SNP effects and corresponding standard errors for selected IVs from the GWASs of both exposure and outcome. Datasets were then harmonized aligning allele information. Primary MR analyses were conducted using the standard inverse‐variance weighted (IVW) method. Generally, the IVW method entails the basic assumption that each SNP is a valid instrumental variable (Burgess et al., 2013). This requires the SNPs to satisfy three assumptions: (i) they are significantly associated with exposure; (ii) they are independent of any confounders; and (iii) they are only associated with the outcome through the exposure. However, the IVW method could lead to bias if any SNP is an invalid instrumental variable. To avoid this, we performed a sensitivity analysis using three additional MR methods. The weighted median method is a median‐based approach that can provide consistent estimates even when up to 50% of the SNPs are invalid instrumental variables (Bowden et al., 2016). MR‐Egger introduces an intercept term to control for the impacts of invalid instrumental variables (Bowden et al., 2015). MR‐PRESSO provided a consistent estimate by detecting and correcting for outliers (Verbanck et al., 2018). Furthermore, we performed leave‐one‐out analyses to evaluate the robustness of the causality by removing potentially influential SNPs. All MR analyses were carried out using the “MendelianRandomization” and “MR‐PRESSO” R packages. Statistical significance was set at a Bonferroni‐corrected threshold of *p* < .016 (0.05/3).

### 2.4 Ethical statement

All GWAS summary statistics were downloaded from the public domain. Therefore, no ethical approval and consent were required for this study.

## 3 RESULTS

### 3.1 Effect infant head circumference on human intelligence

**FIGURE 1:**
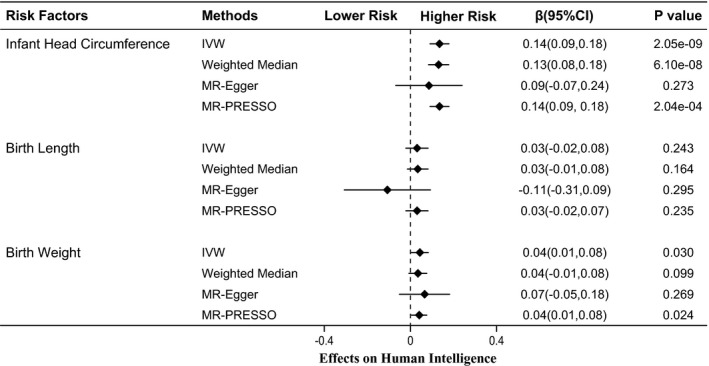
MR associations of birth parameters and intelligence. MR, Mendelian randomization; IVW, inverse‐variance weighted

**FIGURE 2:**
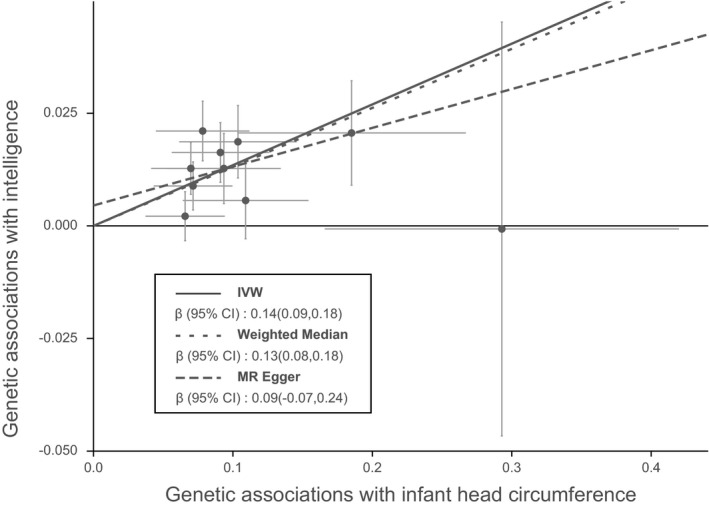
Scatter plot showing the relationship of SNP effects on infant head circumference against SNP effects on intelligence. The slope of each line corresponded to estimated MR effect per method. SNP, single nucleotide polymorphism; MR, Mendelian randomization; IVW, inverse‐variance weighted

Figure 1 shows MR associations between birth parameters and human intelligence. Using 10 SNPs (Table 1) as instrumental variables, we found that 1 standard deviation (1‐*SD*) increase in infant head circumference was associated with 0.14‐fold higher scores in intelligence tests (β = 0.14, 95% confidence interval (CI): 0.09 to 0.18, P_IVW_ = 2.05 × 10^–9^). The weighted median (β = 0.13, 95% CI: 0.08 to 0.18, P_WM_ = 6.10 × 10^–8^) and MR‐PRESSO (β = 0.14, 95% CI: 0.09 to 0.18, P_MR‐PRESSO_ = 2.04 × 10^–4^) analyses showed similar results with the IVW method. Notably, analysis with the MR‐Egger method showed a non‐significant association between infant head circumference and intelligence. This is because the MR‐Egger method is out of work when all variants showed the same direction of causal link (just like in our case in Figure 2) (Bowden et al., 2016). Ignoring the invalid result of MR‐Egger, the causal relationship between infant head circumference and intelligence was robust when sensitivity analysis was performed.

### 3.2 Leave‐one‐out analysis

**FIGURE 3:**
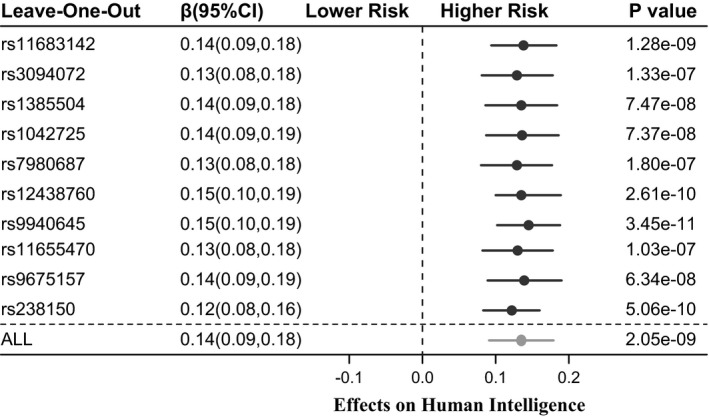
Leave‐one‐out analysis. MR associations were estimated using the IVW method by excluding each SNP in turns. MR, Mendelian randomization; IVW, inverse‐variance weighted; SNP, single nucleotide polymorphism

We also performed leave‐one‐out analyses to identify potential influential SNPs; Figure 3 presents the results of the leave‐one‐out analyses. By sequentially removing each of the 10 SNPs, the MR showed robust association estimates with a fluctuant β estimate ranging from 0.12–0.15. All the results suggested a robust causal relationship between infant head circumference and human intelligence.

### 3.3 Effects of birth length and birth weight on human intelligence

Our study results provided no evidence that indicate the presence of a causal relationship between birth length and intelligence (β = 0.03, 95% CI: −0.02 to 0.08, P_IVW_ = 0.243). These non‐significant results were also observed after statistical analyses (P_WM_ = 0.164; P_MR‐Egger_ = 0.295; P_MR‐PRESSO_ = 0.235). For birth weight, we found that a 1‐*SD* increase in birth weight was associated with better performance in intelligence tests (β = 0.04, 95% CI: 0.01 to 0.08, P_IVW_ = 0.030). However, the statistical P value did not reach the Bonferroni‐corrected threshold of *p* <.016. The MR‐PRESSO method produced a similar result (P_MR‐PRESSO_ = 0.024), but analyses using the weighted median and MR‐Egger methods showed no evidence of association (P_WM_ = 0.099; P_MR‐Egger_ = 0.269). Thus, our study results showed that the genetic determinants of birth weight were not strongly associated with intelligence.

## 4 DISCUSSION

We performed an MR study to investigate the causal effects of infant head circumference, birth length, and birth weight on human intelligence by extracting genetic association datasets from large‐scale GWASs of birth parameters and human intelligence. To the best of our knowledge, this is the first study of its kind. Using 10 SNPs as instrumental variables, our study results indicated the presence of a causal relationship between infant head circumference and intelligence. However, our study provided no evidence to support the existence of causal relationships between birth length, birth weight, and intelligence.

Infant head circumference has long been recognized as a predictor for neurocognitive performance. Bach et al. conducted a nationwide cohort study of 536,921 children in Denmark and found that head circumference at birth was causally related to childhood school performance (Bach et al., 2020). Leppanen et al. found that increase in head circumference between birth and 2 years of age correlated with full‐scale intelligence quotient in non‐small for gestational age children (Leppanen et al., 2014). Jaekel et al. reported that head circumference at birth and head growth in childhood could predict intelligence development from ages 6–26 years in both preterm and term‐born individuals (Jaekel et al., 2019). Our study provided consistent results that indicate the presence of a causal relationship between infant head circumference and intelligence. Furthermore, the MR design of our study prevented the potential limitation caused by confounding environmental factors, which provided stronger evidence of causality between infant head circumference and human intelligence.

The impact of genetic factors should be taken into consideration as they play essential roles in determining the causal relationship between head circumference and intelligence. In the present study, some out of the 10 SNPs used as instrumental variables for infant head circumference (e.g., rs238150, rs7980687, rs3094072 and rs11655470) presented significant associations with intelligence. SBNO1, the corresponding gene of rs7980687, is associated with intellectual disability (Bulayeva et al., 2015). A previous study of zebrafish also showed that knockdown of the SBNO1 gene specifically affects regionalization along the anterior–posterior axis of the brain, suggesting that SBNO1 has essential roles in brain development (Takano et al., 2011). Although the biological functions of these SNPs are unclear, they provide valuable information that advance the understanding of the biological mechanism of differences in human intelligence.

The relationships between birth weight, birth length, and intelligence remain controversial. Several studies suggested that children with lower birth weights have significantly lower cognitive and academic performances later in life than children with normal birth weight (Allotey et al., 2018; Broekman et al., 2009; Goisis et al., 2017; Horta et al., 2017; Leppanen et al., 2014; Pongcharoen et al., 2012); however, some other studies showed no evidence of such associations (Jensen et al., 2015; Shenkin et al., 2009). Similar scenarios were observed for birth length as well (Broekman et al., 2009; Eide et al., 2007; Jensen et al., 2015; Shenkin et al., 2009). Our MR‐based study showed no associations between birth weight, birth length, and intelligence, which might indicate that brain development rather than general fetal growth was responsible for the development of intelligence.

The present study had several strengths. First, our study was implemented using the novel MR study design, which prevents the limitation caused by confounders that are common in observational studies. Second, the MR approach involved the utilization of summary‐level data from publicly available GWAS data sources; this provided a consistent estimate without the usual cost of time, labor, and money that is common in cohort studies. Third, we performed sensitivity analyses using multiple methods, including the weighted median, MR‐Egger, and MR‐PRESSO methods and the leave‐one‐out analysis. Our study provided robust estimates that indicate the presence of causal relationships between birth parameters and human intelligence.

The present study also had some limitations. First, the SNPs for infant head circumference and birth length were selected using a relatively relaxed threshold (*p* < 1×10^−5^); more samples should be collected for future GWASs to generate more genome‐wide significant SNPs. Second, our study captured multiple genetic variants that were associated with both infant head circumference and intelligence; however, their molecular mechanisms needed to be studied further. Finally, the GWASs for birth parameters were performed using quantitative traits, which cannot capture the causal effects of specific birth characters (e.g., premature infant or newborns with very low birth weight) on human intelligence.

## 5 CONCLUSIONS

The present MR study suggests that increase in genetically determined infant head circumference is associated with better performance in intelligence tests and that the causal relationship is independent of environmental factors. However, our results provided no evidence that indicates the presence of causal relationships between birth length, birth weight, and intelligence. These findings might indicate that that brain development rather than general fetal growth was responsible for the development of intelligence.

## CONFLICT OF INTEREST

The authors have no conflicts of interest to disclose.

## AUTHOR CONTRIBUTIONS

LQ and JY conceptualized and designed the study, conducted the data analysis, and drafted the manuscript. FG and BY collected data and carried out the initial analyses. LY and WW contributed to the interpretation of results. LB, XM, and JY critically reviewed and revised the manuscript. All authors approved the final manuscript as submitted and agreed to be accountable for all aspects of the work.

### PEER REVIEW

The peer review history for this article is available at https://publons.com/publon/10.1002/brb3.2183.

## Supporting information

Table S1

## Data Availability

Publicly available datasets are analyzed in this study. The GWAS summary results of infant head circumference, birth length, and birth weight are available at the Early Growth Genetics Consortium (http://egg‐consortium.org/). GWAS dataset of human intelligence is publicly available at the Complex Trait Genetics lab (https://ctg.cncr.nl/).
