# Supplementary material for: Mendelian randomization suggests that head circumference, but not birth weight and length, associates with intelligence
Source: Brain Behav. 2021 May 10;11(6):e02183. doi: 10.1002/brb3.2183 (PMC8213647; doi:10.1002/brb3.2183)
Supplement: Supplementary file 1 — Table S1 [file BRB3-11-e02183-s001.docx]

**Table S1 Genetic predictors of birth length and birth weight and their associations with intelligence**

| **Exposure** | **SNP** | **CHR** | **EA** | **EAF** | **Association with exposure** | | **Association with intelligence** | |
| --- | --- | --- | --- | --- | --- | --- | --- | --- |
|  |  |  |  |  | **β** | **P Value** | **β** | **P Value** |
| Birth Length | rs10185378 | 2 | T | 0.060 | 0.090 | 8.50E-06 | -0.016 | 3.93E-03 |
| Birth Length | rs1042725 | 12 | T | 0.490 | -0.040 | 6.70E-07 | -0.009 | 1.14E-03 |
| Birth Length | rs10483213 | 22 | A | 0.100 | -0.060 | 8.80E-06 | -0.013 | 7.90E-03 |
| Birth Length | rs11037473 | 11 | A | 0.060 | -0.110 | 2.20E-07 | 0.007 | 0.1726 |
| Birth Length | rs11205277 | 1 | A | 0.370 | -0.040 | 8.00E-06 | -0.001 | 0.7788 |
| Birth Length | rs12545524 | 8 | A | 0.140 | -0.080 | 1.50E-08 | -0.007 | 0.0537 |
| Birth Length | rs12967139 | 18 | A | 0.320 | 0.050 | 1.80E-06 | 0.006 | 0.04144 |
| Birth Length | rs13146972 | 4 | T | 0.450 | 0.040 | 4.40E-06 | 0.003 | 0.3633 |
| Birth Length | rs13229771 | 7 | T | 0.130 | 0.070 | 2.80E-06 | 0.007 | 0.1158 |
| Birth Length | rs1576672 | 1 | T | 0.470 | -0.050 | 1.40E-06 | 0.005 | 0.06013 |
| Birth Length | rs16985662 | 22 | A | 0.180 | -0.060 | 8.10E-06 | 0.006 | 0.08123 |
| Birth Length | rs17034876 | 2 | T | 0.300 | 0.050 | 5.30E-06 | 0.002 | 0.7787 |
| Birth Length | rs1984119 | 9 | T | 0.270 | 0.050 | 3.20E-07 | -0.001 | 0.8343 |
| Birth Length | rs2904185 | 4 | T | 0.450 | 0.040 | 8.30E-06 | 0.002 | 0.3936 |
| Birth Length | rs3011890 | 6 | T | 0.270 | -0.050 | 1.60E-06 | -0.003 | 0.6358 |
| Birth Length | rs6570507 | 6 | A | 0.290 | -0.050 | 3.60E-07 | -0.001 | 0.8233 |
| Birth Length | rs6798189 | 3 | A | 0.220 | 0.050 | 1.40E-06 | 0.006 | 0.05456 |
| Birth Length | rs724577 | 4 | A | 0.290 | 0.050 | 4.50E-08 | 0.014 | 2.57E-06 |
| Birth Length | rs740746 | 10 | A | 0.260 | 0.040 | 5.20E-06 | -0.001 | 0.9339 |
| Birth Length | rs905938 | 1 | T | 0.240 | -0.050 | 2.60E-07 | -0.002 | 0.5811 |
| Birth Length | rs9327035 | 5 | T | 0.400 | 0.050 | 2.40E-06 | -0.005 | 0.06453 |
| Birth Weight | rs10883846 | 10 | C | 0.615 | 0.017 | 1.30E-10 | -0.006 | 0.05175 |
| Birth Weight | rs7076938 | 10 | T | 0.735 | 0.032 | 2.10E-28 | -0.001 | 0.8444 |
| Birth Weight | rs4350272 | 10 | A | 0.269 | 0.017 | 3.60E-09 | 0.006 | 0.05506 |
| Birth Weight | rs9645500 | 10 | G | 0.694 | 0.024 | 1.80E-18 | 0.006 | 0.04197 |
| Birth Weight | rs1112718 | 10 | G | 0.404 | 0.026 | 3.80E-23 | -0.001 | 0.7634 |
| Birth Weight | rs4444073 | 11 | A | 0.520 | 0.020 | 2.70E-15 | 0.001 | 0.9153 |
| Birth Weight | rs11042596 | 11 | T | 0.336 | 0.027 | 4.30E-22 | 0.007 | 0.01438 |
| Birth Weight | rs234864 | 11 | A | 0.547 | 0.016 | 1.70E-09 | -0.001 | 0.6205 |
| Birth Weight | rs5030317 | 11 | C | 0.733 | 0.017 | 2.70E-09 | 0.002 | 0.5655 |
| Birth Weight | rs667515 | 11 | G | 0.618 | 0.018 | 9.30E-12 | 0.001 | 0.8447 |
| Birth Weight | rs61885091 | 11 | A | 0.169 | 0.023 | 4.80E-10 | 0.001 | 0.7514 |
| Birth Weight | rs10830963 | 11 | G | 0.277 | 0.019 | 2.80E-11 | 0.001 | 0.9636 |
| Birth Weight | rs2647873 | 12 | A | 0.520 | 0.018 | 2.90E-12 | -0.002 | 0.4944 |
| Birth Weight | rs3184504 | 12 | C | 0.521 | 0.023 | 2.60E-19 | 0.006 | 0.02875 |
| Birth Weight | rs11055030 | 12 | G | 0.718 | 0.020 | 3.90E-12 | -0.006 | 0.03864 |
| Birth Weight | rs2306547 | 12 | C | 0.534 | 0.019 | 4.40E-13 | -0.002 | 0.3862 |
| Birth Weight | rs6582623 | 12 | C | 0.869 | 0.024 | 1.10E-09 | 0.001 | 0.974 |
| Birth Weight | rs7968682 | 12 | G | 0.486 | 0.042 | 4.20E-60 | -0.009 | 1.83E-03 |
| Birth Weight | rs1480470 | 12 | G | 0.631 | 0.024 | 1.40E-19 | 0.001 | 0.7422 |
| Birth Weight | rs9549046 | 13 | A | 0.118 | 0.029 | 8.00E-13 | 0.001 | 0.8167 |
| Birth Weight | rs34217484 | 13 | A | 0.264 | 0.019 | 6.80E-11 | -0.003 | 0.4075 |
| Birth Weight | rs9318511 | 13 | C | 0.873 | 0.027 | 6.00E-12 | -0.003 | 0.4026 |
| Birth Weight | rs6575803 | 14 | C | 0.895 | 0.032 | 1.30E-12 | 0.004 | 0.4096 |
| Birth Weight | rs72681869 | 14 | C | 0.011 | 0.084 | 5.90E-10 | 0.006 | 0.7803 |
| Birth Weight | rs75844534 | 15 | A | 0.124 | 0.026 | 4.90E-11 | 0.008 | 0.08185 |
| Birth Weight | rs339969 | 15 | A | 0.619 | 0.017 | 2.20E-10 | 0.005 | 0.1045 |
| Birth Weight | rs4932373 | 15 | A | 0.680 | 0.020 | 3.00E-13 | -0.006 | 0.03672 |
| Birth Weight | rs55958435 | 15 | A | 0.748 | 0.025 | 1.60E-16 | 0.001 | 0.8882 |
| Birth Weight | rs7402983 | 15 | A | 0.405 | 0.024 | 2.60E-19 | -0.002 | 0.7756 |
| Birth Weight | rs11630479 | 15 | G | 0.703 | 0.014 | 8.90E-07 | -0.003 | 0.2798 |
| Birth Weight | rs2045457 | 16 | G | 0.311 | 0.016 | 6.30E-09 | -0.001 | 0.8975 |
| Birth Weight | rs40434 | 16 | G | 0.391 | 0.017 | 3.00E-10 | 0.003 | 0.2207 |
| Birth Weight | rs28544888 | 16 | C | 0.911 | 0.026 | 1.60E-08 | 0.001 | 0.9556 |
| Birth Weight | rs9909342 | 17 | A | 0.381 | 0.018 | 2.20E-11 | 0.005 | 0.09582 |
| Birth Weight | rs7223535 | 17 | G | 0.732 | 0.021 | 2.10E-13 | -0.009 | 0.00543 |
| Birth Weight | rs11867479 | 17 | T | 0.353 | 0.017 | 1.10E-10 | 0.002 | 0.5479 |
| Birth Weight | rs10221267 | 17 | T | 0.512 | 0.017 | 6.50E-11 | 0.001 | 0.6154 |
| Birth Weight | rs222857 | 17 | T | 0.575 | 0.026 | 1.10E-24 | -0.004 | 0.1615 |
| Birth Weight | rs4511593 | 17 | T | 0.650 | 0.017 | 1.10E-10 | 0.001 | 0.6551 |
| Birth Weight | rs78378222 | 17 | G | 0.013 | 0.079 | 1.80E-11 | -0.003 | 0.8082 |
| Birth Weight | rs73354194 | 17 | C | 0.025 | 0.061 | 1.00E-11 | -0.001 | 0.9614 |
| Birth Weight | rs11082304 | 18 | T | 0.508 | 0.016 | 4.20E-10 | 0.001 | 0.7868 |
| Birth Weight | rs41355649 | 19 | G | 0.934 | 0.034 | 1.20E-10 | -0.001 | 0.9392 |
| Birth Weight | rs1129156 | 19 | T | 0.268 | 0.017 | 2.50E-09 | -0.004 | 0.207 |
| Birth Weight | rs147957154 | 19 | T | 0.132 | 0.023 | 2.80E-09 | -0.001 | 0.8789 |
| Birth Weight | rs2779165 | 19 | G | 0.184 | 0.022 | 7.60E-11 | -0.009 | 0.01821 |
| Birth Weight | rs516246 | 19 | C | 0.506 | 0.018 | 9.30E-12 | 0.006 | 0.02698 |
| Birth Weight | rs147110934 | 19 | G | 0.975 | 0.052 | 1.60E-09 | 0.017 | 0.06701 |
| Birth Weight | rs8106042 | 19 | G | 0.281 | 0.020 | 2.20E-12 | 0.005 | 0.09109 |
| Birth Weight | rs80278614 | 1 | A | 0.054 | 0.040 | 6.50E-12 | -0.002 | 0.7545 |
| Birth Weight | rs905938 | 1 | C | 0.262 | 0.026 | 2.80E-19 | -0.002 | 0.5811 |
| Birth Weight | rs670523 | 1 | G | 0.669 | 0.019 | 7.60E-12 | 0.002 | 0.4116 |
| Birth Weight | rs72480273 | 1 | C | 0.182 | 0.023 | 4.00E-11 | 0.002 | 0.5697 |
| Birth Weight | rs10913200 | 1 | G | 0.972 | 0.051 | 2.00E-10 | -0.016 | 0.1105 |
| Birth Weight | rs61830764 | 1 | A | 0.377 | 0.017 | 1.10E-09 | -0.007 | 0.01508 |
| Birth Weight | rs3806315 | 1 | A | 0.591 | 0.018 | 2.80E-11 | 0.004 | 0.1612 |
| Birth Weight | rs708122 | 1 | C | 0.681 | 0.017 | 2.50E-09 | -0.004 | 0.16 |
| Birth Weight | rs12401656 | 1 | G | 0.865 | 0.025 | 3.40E-11 | -0.002 | 0.6275 |
| Birth Weight | rs1203876 | 20 | C | 0.046 | 0.038 | 9.40E-10 | 0.002 | 0.7471 |
| Birth Weight | rs11698914 | 20 | C | 0.233 | 0.032 | 1.20E-24 | -0.002 | 0.4846 |
| Birth Weight | rs2889874 | 20 | G | 0.452 | 0.016 | 9.40E-10 | 0.001 | 0.636 |
| Birth Weight | rs1012167 | 20 | C | 0.401 | 0.024 | 1.20E-19 | 0.007 | 0.188 |
| Birth Weight | rs753381 | 20 | T | 0.451 | 0.015 | 3.40E-09 | -0.002 | 0.3884 |
| Birth Weight | rs6026449 | 20 | C | 0.627 | 0.017 | 2.50E-10 | -0.002 | 0.5141 |
| Birth Weight | rs73143584 | 20 | A | 0.110 | 0.029 | 1.80E-11 | -0.004 | 0.3694 |
| Birth Weight | rs2229742 | 21 | G | 0.881 | 0.027 | 7.40E-11 | 0.009 | 0.04002 |
| Birth Weight | rs220193 | 21 | A | 0.225 | 0.021 | 4.10E-11 | 0.001 | 0.7077 |
| Birth Weight | rs134594 | 22 | C | 0.351 | 0.017 | 5.80E-10 | -0.002 | 0.5327 |
| Birth Weight | rs41311445 | 22 | A | 0.903 | 0.033 | 3.30E-13 | 0.015 | 1.78E-03 |
| Birth Weight | rs7285579 | 22 | C | 0.698 | 0.017 | 2.70E-09 | -0.002 | 0.5538 |
| Birth Weight | rs11096402 | 23 | G | 0.247 | 0.021 | 2.50E-14 | 0.004 | 0.2827 |
| Birth Weight | rs7067170 | 23 | G | 0.760 | 0.017 | 3.80E-10 | -0.009 | 0.01153 |
| Birth Weight | rs7886910 | 23 | C | 0.934 | 0.033 | 2.80E-12 | -0.009 | 0.2146 |
| Birth Weight | rs2280235 | 2 | G | 0.259 | 0.018 | 6.90E-10 | -0.001 | 0.6687 |
| Birth Weight | rs10181515 | 2 | T | 0.225 | 0.021 | 2.10E-12 | -0.005 | 0.1252 |
| Birth Weight | rs2551347 | 2 | T | 0.749 | 0.024 | 1.90E-16 | 0.006 | 0.04792 |
| Birth Weight | rs754868 | 2 | G | 0.419 | 0.016 | 6.70E-10 | -0.006 | 0.03203 |
| Birth Weight | rs17034876 | 2 | T | 0.700 | 0.042 | 3.10E-47 | 0.002 | 0.7787 |
| Birth Weight | rs4953353 | 2 | G | 0.632 | 0.018 | 3.50E-11 | 0.005 | 0.3268 |
| Birth Weight | rs10495563 | 2 | A | 0.664 | 0.022 | 2.10E-16 | 0.001 | 0.9457 |
| Birth Weight | rs11708067 | 3 | G | 0.238 | 0.041 | 1.60E-42 | -0.005 | 0.1545 |
| Birth Weight | rs2306700 | 3 | T | 0.136 | 0.023 | 1.80E-09 | 0.01 | 0.01304 |
| Birth Weight | rs10935733 | 3 | T | 0.399 | 0.019 | 2.30E-13 | -0.005 | 0.07322 |
| Birth Weight | rs1482852 | 3 | A | 0.599 | 0.050 | 1.60E-82 | -0.01 | 3.69E-04 |
| Birth Weight | rs11711420 | 3 | T | 0.747 | 0.019 | 3.20E-10 | 0.003 | 0.3924 |
| Birth Weight | rs2168443 | 3 | T | 0.379 | 0.017 | 3.90E-10 | -0.003 | 0.3378 |
| Birth Weight | rs6533183 | 4 | C | 0.352 | 0.022 | 6.80E-16 | -0.018 | 9.94E-11 |
| Birth Weight | rs116807401 | 4 | C | 0.018 | 0.077 | 2.20E-13 | 0.004 | 0.7948 |
| Birth Weight | rs6845999 | 4 | T | 0.431 | 0.026 | 1.50E-24 | 0.002 | 0.407 |
| Birth Weight | rs4144829 | 4 | C | 0.267 | 0.036 | 4.30E-34 | -0.014 | 3.64E-06 |
| Birth Weight | rs1981627 | 5 | G | 0.585 | 0.017 | 8.40E-11 | -0.001 | 0.7435 |
| Birth Weight | rs2946179 | 5 | C | 0.734 | 0.020 | 1.10E-11 | 0.004 | 0.1852 |
| Birth Weight | rs1818782 | 5 | C | 0.637 | 0.016 | 4.20E-09 | -0.006 | 0.02712 |
| Birth Weight | rs351930 | 5 | T | 0.801 | 0.019 | 2.90E-09 | 0.001 | 0.8229 |
| Birth Weight | rs854037 | 5 | A | 0.814 | 0.027 | 9.40E-16 | 0.014 | 5.37E-05 |
| Birth Weight | rs28365970 | 5 | C | 0.741 | 0.020 | 1.70E-11 | 0.004 | 0.2018 |
| Birth Weight | rs76094073 | 6 | G | 0.121 | 0.027 | 1.60E-11 | -0.006 | 0.1374 |
| Birth Weight | rs6925689 | 6 | T | 0.494 | 0.015 | 6.40E-09 | -0.014 | 2.82E-07 |
| Birth Weight | rs6569647 | 6 | T | 0.802 | 0.020 | 6.30E-10 | 0.005 | 0.1534 |
| Birth Weight | rs6930558 | 6 | T | 0.747 | 0.022 | 3.40E-13 | -0.002 | 0.6001 |
| Birth Weight | rs962554 | 6 | T | 0.715 | 0.017 | 3.80E-09 | 0.002 | 0.5747 |
| Birth Weight | rs10872678 | 6 | T | 0.724 | 0.032 | 9.80E-29 | 0.003 | 0.3119 |
| Birth Weight | rs2934844 | 6 | T | 0.672 | 0.021 | 1.80E-13 | -0.004 | 0.2428 |
| Birth Weight | rs35261542 | 6 | C | 0.733 | 0.041 | 2.80E-45 | 0.006 | 0.05645 |
| Birth Weight | rs9379832 | 6 | A | 0.730 | 0.022 | 1.10E-13 | -0.002 | 0.5191 |
| Birth Weight | rs9366778 | 6 | G | 0.627 | 0.018 | 2.90E-11 | 0.002 | 0.4815 |
| Birth Weight | rs9267812 | 6 | T | 0.133 | 0.023 | 3.10E-09 | -0.006 | 0.1224 |
| Birth Weight | rs1547669 | 6 | G | 0.497 | 0.018 | 6.20E-12 | -0.001 | 0.8563 |
| Birth Weight | rs75104038 | 6 | A | 0.060 | 0.045 | 4.30E-16 | 0.008 | 0.1964 |
| Birth Weight | rs9348981 | 6 | T | 0.710 | 0.021 | 2.20E-13 | -0.003 | 0.2948 |
| Birth Weight | rs7744700 | 6 | T | 0.711 | 0.020 | 1.60E-11 | -0.001 | 0.9444 |
| Birth Weight | rs6467157 | 7 | T | 0.713 | 0.020 | 1.50E-11 | -0.019 | 1.15E-03 |
| Birth Weight | rs59084784 | 7 | A | 0.323 | 0.017 | 2.40E-09 | 0.004 | 0.204 |
| Birth Weight | rs34776209 | 7 | C | 0.755 | 0.023 | 8.50E-15 | -0.009 | 0.00543 |
| Birth Weight | rs4719648 | 7 | C | 0.577 | 0.019 | 2.60E-13 | 0.002 | 0.5001 |
| Birth Weight | rs138715366 | 7 | C | 0.991 | 0.240 | 4.00E-61 | -0.024 | 0.2213 |
| Birth Weight | rs11983722 | 7 | A | 0.938 | 0.032 | 3.10E-09 | -0.001 | 0.898 |
| Birth Weight | rs10265057 | 7 | G | 0.092 | 0.027 | 1.30E-09 | -0.009 | 0.04345 |
| Birth Weight | rs2237467 | 7 | A | 0.221 | 0.018 | 5.30E-09 | 0.012 | 2.78E-04 |
| Birth Weight | rs112139215 | 7 | A | 0.068 | 0.047 | 2.80E-20 | -0.005 | 0.334 |
| Birth Weight | rs2282978 | 7 | C | 0.326 | 0.018 | 1.70E-11 | 0.005 | 0.114 |
| Birth Weight | rs7819593 | 8 | C | 0.243 | 0.022 | 6.20E-13 | 0.003 | 0.3234 |
| Birth Weight | rs10283100 | 8 | G | 0.946 | 0.042 | 7.00E-13 | -0.003 | 0.6558 |
| Birth Weight | rs13271368 | 8 | C | 0.761 | 0.020 | 2.30E-11 | -0.011 | 9.70E-04 |
| Birth Weight | rs13257363 | 8 | G | 0.591 | 0.018 | 2.00E-11 | 0.006 | 0.02555 |
| Birth Weight | rs9657468 | 8 | G | 0.334 | 0.015 | 7.90E-08 | -0.003 | 0.3041 |
| Birth Weight | rs732563 | 8 | C | 0.504 | 0.017 | 1.30E-11 | 0.001 | 0.644 |
| Birth Weight | rs34036147 | 8 | T | 0.688 | 0.018 | 8.40E-11 | -0.006 | 0.05294 |
| Birth Weight | rs13266210 | 8 | A | 0.786 | 0.027 | 1.50E-17 | -0.002 | 0.4938 |
| Birth Weight | rs72656010 | 8 | T | 0.868 | 0.028 | 1.40E-13 | 0.002 | 0.5841 |
| Birth Weight | rs62496903 | 8 | T | 0.083 | 0.033 | 6.70E-12 | 0.005 | 0.4153 |
| Birth Weight | rs2418135 | 9 | A | 0.522 | 0.020 | 1.50E-14 | 0.001 | 0.8767 |
| Birth Weight | rs1323438 | 9 | C | 0.718 | 0.019 | 5.60E-11 | -0.006 | 0.06893 |
| Birth Weight | rs3933326 | 9 | G | 0.676 | 0.021 | 2.30E-14 | -0.003 | 0.6511 |
| Birth Weight | rs10985827 | 9 | G | 0.141 | 0.030 | 6.10E-16 | -0.003 | 0.3787 |
| Birth Weight | rs28505901 | 9 | A | 0.249 | 0.024 | 2.50E-15 | 0.004 | 0.2776 |
| Birth Weight | rs7854962 | 9 | C | 0.785 | 0.022 | 1.00E-11 | 0.009 | 0.01182 |
| Birth Weight | rs28457693 | 9 | G | 0.109 | 0.044 | 9.90E-26 | -0.016 | 1.20E-04 |
| Birth Weight | rs71486610 | 10 | C | 0.477 | 0.020 | 3.20E-15 | Not available | Not available |
| Birth Weight | rs2274224 | 10 | C | 0.434 | 0.021 | 9.80E-17 | Not available | Not available |
| Birth Weight | rs76895963 | 12 | G | 0.021 | 0.076 | 2.50E-13 | Not available | Not available |
| Birth Weight | rs255773 | 19 | C | 0.536 | 0.018 | 1.30E-11 | Not available | Not available |
| Birth Weight | rs6040076 | 20 | C | 0.500 | 0.019 | 4.40E-13 | Not available | Not available |
| Birth Weight | rs6033062 | 20 | A | 0.460 | 0.016 | 5.20E-10 | Not available | Not available |
| Birth Weight | rs56188432 | 2 | G | 0.002 | 0.258 | 1.40E-16 | Not available | Not available |
| Birth Weight | rs186606513 | 2 | G | 0.978 | 0.061 | 2.70E-09 | Not available | Not available |
